# Modulatory Effects of Polyphenols on Apoptosis Induction: Relevance for Cancer Prevention

**DOI:** 10.3390/ijms9030213

**Published:** 2008-02-28

**Authors:** Massimo D'Archivio, Carmela Santangelo, Beatrice Scazzocchio, Rosaria Varì, Carmela Filesi, Roberta Masella, Claudio Giovannini

**Affiliations:** National Centre for Food Quality and Risk Assessment, Istituto Superiore di Sanità, Viale Regina Elena 299, 00161 Rome Italy

**Keywords:** polyphenols, carcinogenesis, apoptosis

## Abstract

Polyphenols, occurring in fruit and vegetables, wine, tea, extra virgin olive oil, chocolate and other cocoa products, have been demonstrated to have clear antioxidant properties *in vitro*, and many of their biological actions have been attributed to their intrinsic reducing capabilities. However, it has become clear that, in complex biological systems, polyphenols exhibit several additional properties which are yet poorly understood. Apoptosis is a genetically controlled and evolutionarily conserved form of cell death of critical importance for the normal embryonic development and for the maintenance of tissue homeostasis in the adult organism. The malfunction of the death machinery may play a primary role in various pathological processes, since too little or too much apoptosis can lead to proliferative or degenerative diseases, respectively. Cancer cells are characterized by a deregulated proliferation, and/or an inability to undergo programmed cell death. A large body of evidence indicates that polyphenols can exert chemopreventive effects towards different organ specific cancers, affecting the overall process of carcinogenesis by several mechanisms: inhibition of DNA synthesis, modulation of ROS production, regulation of cell cycle arrest, modulation of survival/proliferation pathways. In addition, polyphenols can directly influence different points of the apoptotic process, and/or the expression of regulatory proteins. Although the bulk of data has been obtained in *in vitro* systems, a number of clinical studies suggesting a preventive and therapeutic effectiveness of polyphenols *in vivo* is available. However, a deeper knowledge of the underlying mechanisms responsible for the modulation of apoptosis by polyphenols, and their real effectiveness, is necessary in order to propose them as potential chemopreventive and chemotherapeutic candidates for cancer treatment.

## 1. Introduction

Polyphenols are the most abundant antioxidants in human diet and are widespread constituents of fruit, vegetables, cereals, extra virgin olive oil, dry legumes, chocolate and beverages, such as tea, coffee and wine [1]. Despite their wide distribution, the healthy effects of dietary polyphenols have come to the attention of nutritionists only in the last years. One of the main factors responsible for delayed research on polyphenols is the diversity and the complexity of their chemical structure which influences the antioxidant power. As antioxidants, polyphenols may protect cell constituents against oxidative damage. Therefore, they can limit the risk of various degenerative diseases associated with oxidative stress, such as cardiovascular diseases, type 2 diabetes and cancer [2–4]. However, accumulating evidence [5–7] indicates that, in complex biological systems, polyphenols exhibit several additional properties which may be independent of conventional antioxidant activities. This is also suggested by at least two considerations. Firstly, phenolic compounds are metabolized *in vivo*, giving rise to compounds that lose the original antioxidant potential [8,9]. Secondly, the concentrations of polyphenols and their metabolites, in plasma or tissues, are lower than those of other antioxidants such as ascorbic acid and α-tocopherol, which renders their competition unlikely [10–13]. Such novel mechanisms of action of dietary polyphenols might entail their interaction with cell signalling consequently influencing gene expression and modulating several cell activities [14].

Dietary polyphenols have attracted a great deal of interest because of their perceived ability to act as highly effective chemopreventive agents [15–17]. In addition, the low toxicity and the very few adverse side effects linked to polyphenols consumption give them potential advantages with respect to the traditional chemotherapeutic agents [18].

Several well-accepted mechanisms may, at least partially, explain the effectiveness of these compounds as chemopreventive agents in cancer cells. In fact they are able to: 1. suppress the over-expression of pro-oxidant enzymes implicated in cancer development; 2. inhibit the transcription factor activation, thus regulating target genes involved in cell survival and proliferation; 3. induce apoptosis; 4. inhibit matrix metalloproteinases (MMPs) and vascular endothelial growth factor (VEGF) counteracting angiogenesis which is involved in metastasis development [17,19–26].

The present review will focus the molecular basis of chemopreventive activity of polyphenols, addressing to their effects on the induction of apoptosis in cancer cells.

## 2. Apoptosis and cancer

Apoptosis is a genetically controlled and evolutionarily conserved form of cell death of critical importance for the normal embryonic development and for the maintenance of tissue homeostasis in the adult organism. Cancer cells are characterized by a deregulated proliferation, and/or an inability to undergo programmed cell death. The machinery responsible for killing and degradation of the cell *via* apoptosis is constitutively expressed and becomes activated through various stimuli.

**Figure 1.**
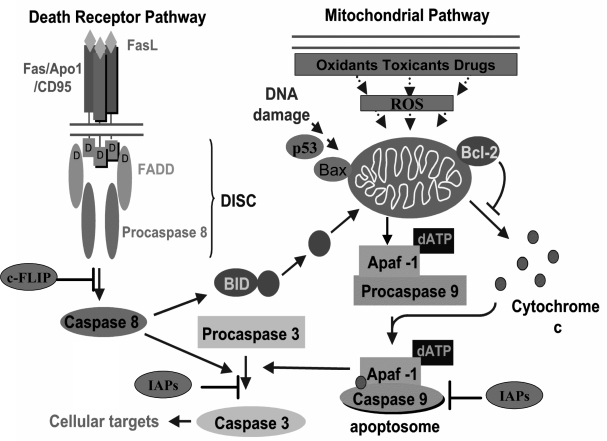
**Apoptosis pathways:** The extrinsic or death receptor pathway (left) is triggered by members of the death receptor superfamily such as Fas. Binding of Fas-L to Fas induces trimerization of the receptor, recruitment of specific adaptor proteins (FADD) and consequently recruitment of procaspase 8 molecules. The multi-molecular complex (DISC) results in the activation of caspase-8, which can be blocked by c-FLIP. Active caspase-8 can in turn activate Bid, a pro-apoptotic member of Bcl-2 family proteins, which represents a crosstalk between extrinsic and intrinsic pathways. Oxidants, toxicants, drugs or ionizing radiation, which all induce ROS overproduction and the stress signalling, can activate the intrinsic pathway (right). The intrinsic or mitochondrial pathway is also triggered by DNA damage via p53 activities. The death stimuli result in loss of mitochondrial membrane integrity and release of cythocrome *c*, Apaf-1 and other pro-apoptotic factors in the cytoplasm. Maintenance or perturbation of mitochondrial membrane potential depends on the ratio between pro-apoptotic (Bax) and anti-apoptotic (Bcl-2) members of Bcl-2 family, by causing or preventing *cythocrome c* release. Multiple molecules of cythocrome *c*, Apaf-1, dATP and procaspase-9 associate to form a supramolecular complex termed ‘apoptosome’, that activates caspase-9 through autocatalysis. Both the activated caspase-9 and caspase-8 cleave procaspase-3 generating the active caspase-3 that, in turn, activates other executor caspases and cleaves cellular targets. Caspase activity is controlled by Inhibitors of Apoptosis Protein (IAPs) family.

Apoptosis, characterized by a set of morphologic changes, can occur in mammalian cells by the extrinsic or intrinsic pathways [27]. The extrinsic or death receptor pathway is activated when a specific ligand binds its corresponding cell-surface death receptor, such as tumour necrosis factor (TNF) receptor, TNF-related apoptosis-inducing ligand (TRAIL) receptor and Fas receptor. In particular, the well-characterized Fas receptor (also called APO-1or CD95) is activated by binding Fas ligand that leads to its trimerization and to the recruitment of Fas-Associated protein with Death Domain (FADD). The consequent conformational changes result in the binding of procaspases-8 to a supramolecular complex called Death-Inducing Signalling Complex (DISC) [28]. Caspase-8 activation can be blocked by cellular FADD-Like interleukin-1ß-converting enzyme Inhibitory Protein (c-FLIP). Conversely, caspase-8 can also activate Bcl-2 interacting domain (Bid), a pro-apoptotic member of the Bcl-2 family of proteins, described below, which, in turn, can directly affect the mitochondrial membrane potential, thus interacting with the intrinsic pathway (Figure 1).

The intrinsic or mitochondrial pathway is activated by different agents, such as oxidants, toxicants, drugs or ionizing radiations, all of which induce ROS overproduction and the onset of oxidative stress. The activation of this pathway is accompanied by the translocation of cytochrome *c* from the mitochondrial intermembrane space into the cytoplasm. Cytochrome *c* and Apoptotic protease-activating factor 1 (Apaf-1), are released from mitochondria and function as proapoptotic factors. Cytochrome *c*, Apaf-1, dATP and procaspase-9 form a supramolecular complex termed ‘apoptosome’, that activates caspase-9 through autocatalysis.

Both the pathways lead to the activation of caspase-3 that, in turn, activates other executor caspases, cleaves cytoskeleton and activates specific DNAses (Figure 1).

Among the molecules that exert their regulatory effect in determining cell fate, the proteins of the Bcl-2 family represent important checkpoints which control the main steps of the apoptotic cell death [29]. The maintenance or perturbation of mitochondrial membrane potential depends, in fact, on the ratio between pro-apoptotic (Bax, Bad, Bak, Bid, Bcl-Xs) and anti-apoptotic (Bcl-2, Bcl-XL, Bag-1, Bcl-W) members of Bcl-2 family [30,31].

Another key protein is the transcription factor p53, also known as tumour protein 53, that regulates cell cycle and apoptosis and hence functions as a tumour suppressor. In the presence of DNA damage, p53 protein arrests cell cycle allowing time for cells to repair DNA. When the damage cannot be successfully repaired, p53 acts as pro-apoptotic signal. The loss of p53, or its mutations, decreases caspase activation and therefore the occurrence of apoptosis. p53 down-regulates several anti-apoptotic genes and/or can directly activate the apoptotic pathways [32–35]. In addition p53 can displace Bax or Bid from pre-existing complexes with Bcl-XL, by binding to Bcl-XL itself, thus triggering apoptosis [36].

## 3. Carcinogenesis and modulation of apoptosis by polyphenols

Carcinogenesis is the result of an imbalance of the tissue homeostasis. In a stable mature tissue the rates of replication and cell death are balanced. In certain circumstances, the sustained rate of cell replication can exceed the rate of apoptosis, resulting in hyperplasia which in itself does not imply tumour development. In fact, cell proliferation is regulated by checkpoint molecules at the major stages of the cell cycle. If anyone of these checkpoints is overruled, cell can be prone to natural or induced mutations. Apoptosis eliminates genetically damaged cells or cells that may be inappropriately induced to proliferate, representing thus a protective mechanism against neoplastic transformation and development of tumours. The mutated cells which escape the apoptotic control can, in fact, become the progeny of neoplastic cell population. The multistage process of carcinogenesis could be divided in three main stages: initiation, promotion and progression [18,37]. In the initiation stage cells can opposite to carcinogens by the activation of different detoxifying enzymes such as the phase I and II enzymes. However, phase I enzymes (e.g. cytocrome P450), by reacting with carcinogens or xenobiotics, form potent electrophile, mutagenic compounds which are able to interact with the DNA triggering, in turn, nucleic acid damage and mutations. Although phase II enzymes (e.g. glutathione transferase) can detoxify these compounds by forming water-soluble glutathione or sulfate conjugates which are easily eliminated by the body, this defence mechanism is often inadequate. The stage of tumour promotion is characterized by cell proliferation which is induced by the activation and/or over-expression of enzymes involved in the synthesis of nucleotides and DNA (ornithine decarboxylase), and in the regulation of the differentiation process (DNA polymerase or topoisomerase II). Moreover, during the promotion stage, ROS overproduction occurs, mainly due to the over-expression of pro-oxidant enzymes (e.g. cyclo-oxygenase, lypoxygenase), which leads to cell damages and further DNA mutations. In the progression stage, the final stage of carcinogenesis, the mutated cells proliferate in uncontrolled manner and acquire a metastatic potential.

Polyphenols can affect the overall process of carcinogenesis by several mechanisms. In particular, polyphenols contribute to counteract oxidative stress occurrence [38,39] and, in so doing, they can contribute to the prevention of cancer onset and development. In fact by modulating oxidative stress in cancer cells, polyphenols can affect the signal transduction, the activation of redox-sensitive transcription factors and the expression of specific genes that influence cell proliferation and apoptosis [40,41]. In addition, a growing body of evidence indicates that polyphenols can directly modulate different points of the apoptotic process and/or the expression of regulatory proteins, such as the release of cytochrome *c* with subsequent activation of caspases-9 and caspases-3 [42–45], the increase of caspases-8 and t-Bid levels [44], the down-regulation of Bcl-2 and Bcl-XL expression, the enhanced expression of Bax and Bak [44,46,47] and the modulation of transcription factor NF-κB [48]. Among the food-derived polyphenols screened for the chemopreventive effectiveness, resveratrol, a stilbene present in high amounts in grapes [49], is particularly interesting because of its ability to affect a broad range of intracellular mediators involved in the initiation, promotion and progression of cancer [50–52].

Resveratrol could prevent or delay the onset of various cancers because of its ability to regulate multiple cellular events associated with carcinogenesis. In particular, it inhibits cell proliferation and induces apoptosis [25]. The induction of apoptosis by resveratrol has been reported to be associated with increased caspase activity [53–56], cell cycle dysregulation [57–59], decreased Bcl-2 and Bcl-XL levels, and increased Bax levels [56,60]. Interestingly, these pro-apoptotic actions have been reported to be frequently associated with the activation of p53 in different cancer cells [60–62]. A recent paper, which reviewed in detail the most convincing studies on resveratrol, pointed out this compound as a promising candidate for chemoprevention and chemotherapy [63]. However, the efficacy of resveratrol is still debated because of the multiplicity of affected targets and the contradictory effects related to the dose and the time of treatment as well as to the cellular phenotype.

The anticarcinogenic activity of tea polyphenols has been shown *in vitro* in different cancer cells (skin, prostate, colon, breast and lung) [26,64]. Green and black tea polyphenols, such as the flavonoid epigallocatechin-3-gallate (EGCG), have been demonstrated to regulate cancer cell growth and transformation, apoptosis, angiogenesis and metastasis, through the regulation of multiple signalling pathways probably depending on the cell type. Specifically, EGCG influences the expression of members of the Bcl-2 family of proteins, as well as of VEGF, MMPs and cell cycle inhibitors [26,65,66]. The hydroxybenzoic acid protocatechuic acid, one of the main metabolites of anthocyanins [67], also found in olives [68], brown rice [69] and tea [70], has been recently shown to induce apoptosis in human gastric adenocarcinoma cells through the Fas/FasL pathway, by activating JNK/p38 kinases [71]. Caffeic acid, a hydroxycinnamic acid found in many types of fruit and in high concentration in coffee [1], induced apoptosis in human breast cancer cells [72] by activating pro-apoptotic factors such as Fas, Bax and caspases. Likewise, it increased caspase-3 activity in stomach cancer, colon cancer and pro-myelocytic leukemia cells [73]. Furthermore, the treatment of glioma cells with caffeic acid induced the release of cytocrome *c* from mitochondria into cytosol and enhanced the expression of p53, Bax and Bak [74]. Pro-apoptotic activity, mediated by caspase-3 dependent mechanism, has been observed also in oral squamous carcinoma cells exposed to different phenolic compounds derived from ginger, a common condiment for various foods and beverages [75,76]. In Jurkat human T-cell leukemia cells, similar compounds activated mitochondrial pathway and altered the balance between the pro-and anti-apoptotic proteins, down regulating the anti-apoptotic Bcl-2 protein and enhancing the expression of the pro-apoptotic Bax [77]. Also curcumin, a polyphenolic compound derived from the rhizome of the plant *Curcuma longa*, induced apoptosis by suppressing the constitutive expression of Bcl-2 and Bcl-XL, and by activating caspase-7 and caspase-9 in mantle cell lymphoma [78] and multiple myeloma [79] cell lines. Recently it has been demonstrated that curcumin induced apoptosis in prostate cancer cells, by down-regulating the expression of Bcl-2 and Bcl-XL and up-regulating the expression of p53, Bax, Bak, and Bim [80].

As mentioned above, cancer cells constitutively generate large, but tolerable, amounts of ROS. Consequently, this suggests that a certain level of oxidative stress may be required to maintain a balance between proliferation and apoptosis [81]. ROS apparently function as signalling molecules in the mitogen-activated protein kinase (MAPKs) pathways [82] leading to the activation of redox-sensitive transcription factors and responsive genes which are involved in the survival and proliferation of cancer cells. The MAPK signalling cascades include extracellular signal-related protein kinases (ERKs), JNKs/stress-activated protein kinases (SAPKs), and p38 kinases [83]. The ERK pathway has been associated with the regulation of cell proliferation since it transmits signals initiated by growth promoters, and may ultimately foster cell growth and survival [15]. In contrast, the activation of JNK and p38 kinases is controlled by stress signalling, such as oxidative stress, and has been associated with the induction of apoptosis [84,85]. The balance between ERK and JNK activation is a key factor for cell survival since both, the decrease of ERK and the increase of JNK, are required to induce apoptosis. The activated MAPKs translocate to the nucleus, where they phosphorylate a number of substrates, including the transcription factors AP-1 and NF-κB which are linked to carcinogenesis and tumour promotion [15]. The activation of AP-1 and NF-κB promotes, in fact, survival and cellular proliferation, while their down-regulation sensitizes cells to apoptosis [81]. The excess of ROS can be scavenged by phenolic compounds. Consequently, the oxidative stress-responsive genes can be suppressed and cancer cell proliferation inhibited. On the other hand, polyphenols can also induce the formation of ROS to achieve an intolerable level of oxidative stress in cancer cells [81]. When the critical threshold for cancer cells to cope with oxidative stress has been reached, key cellular components, such as DNA, are irreparably damaged. In addition, genes involved in initiating cell cycle arrest and/or apoptosis are activated. Therefore polyphenols can either scavenge the constitutive ROS or, paradoxically, generate additional amounts of ROS to inhibit the proliferation of cancer cells. Both the mechanism of action seem to be strictly linked to the phenolic concentration and the experimental conditions. It has been observed, in fact, that low or high concentrations of the same phenolic compound are responsible for antioxidant and pro-oxidant activity, respectively [81,86]. Several studies suggest that polyphenols can scavenge the constitutively high amounts of H_2_O_2_ in different cancer cells, such as human epidermal keratinocytes, U-937 cells, Jurkat cells, HeLa cells and glioma cells [87–89]. Consequently, they were able to block the MAPK signalling, the activation of NF-κB and AP-1, and the induction of responsive genes that stimulate cancer cell proliferation [88]. In particular, resveratrol prevented NF-κB activation induced by phorbol myristate acetate, lipopolysaccaride, okadaic acid, ceramide and, most importantly, H_2_O_2_. Resveratrol had similar effects on the events which lead to the activation of transcription factors, as AP-1 in HeLa cells exposed to either PMA or ultraviolet radiation [88]. The flavone apigenin, abundantly present in fruit and vegetables, induced growth inhibition of human anaplastic thyroid carcinoma cells, probably by directly inhibiting the phosphorylation of MAPK, or alternatively, by scavenging H_2_O_2_ that activates the protein kinases [90].

On the other hand, polyphenols such as EGCG, quercetin, and gallic acid, can have pro-oxidant effects generating H_2_O_2_ in a time- and concentration-dependent manner when added to cell culture media consequently provoking stressful and/or cytotoxic effects [91]. Likewise, the apoptosis induced in Ha-ras gene-transformed human bronchial epithelial cells by a 24hr-treatment with 25 μM EGCG, or related tea catechins [92], was attributed to ROS production. In fact, the catechins induced the formation of H_2_O_2_ and the addition of catalase prevented the apoptosis. In addition, the tea catechins decreased c-jun protein phosphorylation, and consequently AP-1 activity needed to activate some genes which promote cancer cell viability. Similarly, the apoptosis induced in human oral squamous carcinoma cells by EGCG was attributed to the generation of H_2_O_2_ in the cell culture medium [93]. Finally, it has been recently demonstrated that the 3,4 dihydroxybenzoic acid induced apoptosis in human gastric carcinoma cells by ROS overproduction which is able to activate JNK/p38 MAPKs [94].

It is worth of note that cancer cells, compared to normal cells, are more susceptible to be killed by anticancer drugs and polyphenols as well. This is probably because cancer cells are already close to a threshold for tolerating ROS. In fact, by using the same concentration, phenolic compounds induced apoptosis in cultured cancer cells, but not in their normal counterparts [95–98]. In agreement with these findings, internucleosomal DNA fragmentation was detected in A431 (human epidermoid carcinoma cells), HaCaT (human carcinoma keratinocytes), DU145 (human prostate carcinoma cell line) and L5178Y cell lines (mouse lymphoma cells), but not in normal human epidermal keratinocytes [99] after treatment with EGCG. In addition Hsu et al. [100] demonstrated the influence of the age of the cells studied (differentiated / undifferentiated) by showing that the ECGC induced differentiation of immature normal human keratinocytes after 24 h of treatment, while stimulate cellular proliferation was stimulated in aged keratinocytes (15–25 days). It should be also taken in account that polyphenols can elicit different cellular responses depending on concentration used for the experiment. The dose-dependent effect of EGCG on the inhibition of cell proliferation was demonstrated in neuroblastoma SH-SY5Y cells, where low flavanol concentration (1 μmol/L) induced an anti-apoptotic response, while higher concentration (50 μmol/L) caused a pro-apoptotic effect [101]. In addition EGCG enhanced proliferation at 0.5 μmol/L and did not affect cell growth at 50 μmol/L in normal keratinocytes, while it decreased cell proliferation at both concentrations in squamous carcinoma cells [95]. In conclusion, EGCG seems to possess a dual mechanism of action depending on the concentration, although it appears that this flavanol exerts its action in a selective manner in normal and cancer cells.

## 4. Conclusion

Dietary polyphenols have attracted a great deal of interest because of their perceived ability to act as highly effective chemopreventive and chemotherapeutic agents. In fact they are able to affect the overall process of carcinogenesis by suppressing the over-expression of pro-oxidant enzymes, by inhibiting targets genes involved in cell proliferation and by inducing apoptosis.

Apoptosis represents a protective mechanism against neoplastic transformation and development of tumours by eliminating genetically damaged cells or cells that may be inappropriately induced to proliferate by mitogenic and proliferative stimuli.

A growing body of evidence provides new insight in the comprehension of the cellular and molecular mechanisms responsible for the induction of apoptosis by polyphenols. The experimental data suggest a multifaceted action of polyphenols on the modulation of cell signals and biochemical pathways involved in cell survival and cell death. Moreover these effects depend on the concentration of polyphenols, the cell system, the cell age and the type or stage of the degenerative process.

Although some studies well address the effectiveness of polyphenols in humans [102–104], in our opinion a deeper knowledge of the mechanisms responsible for the induction of apoptosis by polyphenols, and their real effectiveness *in vivo*, is necessary in order to propose them as potential chemopreventive and chemotherapeutic candidates for cancer treatment.
